# Effects of fresh and fermented olive oil solid waste on nitrogen use efficiency in wheat: A dataset

**DOI:** 10.1016/j.dib.2025.111742

**Published:** 2025-05-30

**Authors:** Abd Al Karim Jaafar, Akram Al-Balkhi, Andrés Rodríguez-Seijo

**Affiliations:** a Department of Soil Science, Faculty of Agricultural, Damascus University, Damascus, Syrian Arab Republic; b Department of Plant Biology and Soil Science, Área de Edafoloxía e Química Agrícola, Facultade de Ciencias, Universidade de Vigo, Ourense 32004, Spain; c Instituto de Agroecoloxía e Alimentación (IAA), Universidade de Vigo Campus Auga, Ourense 32004, Spain

**Keywords:** By-product, Nitrogen efficient use, Olive oil solid waste, Organic fertilization, Waste valorisation

## Abstract

Olive Oil Solid Waste (OSW) is a valuable agricultural by-product that can be used as a source of organic matter and nutrients for crops. However, the efficiency of OSW in providing nitrogen to plants depends on its decomposition rate and how nitrogen is available. Here, we investigated the nitrogen utilization coefficient (NUC) from fresh and fermented OSW added to calcareous soil planted with durum wheat (Triticum durum cv Sham 3). Our data showed that the NUC of fermented OSW was significantly higher than that of fresh OSW, with values of 50.15 % and 35.36 %, respectively. This difference was attributed to the enhanced biodegradation of fermented OSW, which resulted in the release of more nitrogen in a form that was readily available for plants.

Specifications Table

**Table:** 

Subject	Agronomy and crop science
Specific subject area	Effect of olive oil wastes subproducts applications on nitrogen utilization coefficient by wheat
Type of data	Tables. Raw and analysed data
Data collection	Experimental assay to assess the impact of different treated olive wastes on plant growth
Data source location	Damascus University
Data accessibility	Mendeley Data https://data.mendeley.com/datasets/2cf9h5d8tf/1
Related research article	None

## 1 Value of the Data

• Fermented Olive solid wastes (OSW) is a more efficient source of nitrogen for plant cultivation than fresh OSW
• The treatment of olive wastes, such as fermentation, should be considered before their application to the field.

## 2 Background

The olive crop in Syria is significant, covering approximately 600,000 hectares with 80 million trees, constituting 10 % of cultivated land and 60 % of fruit tree areas [1]. During olive oil production, 35–80 kg of olive residues are generated per 100 kg of olives, forming olive oil solid waste (OSW) [2,3]. This agro-industrial waste, rich in organic matter (94 %) and nutrients like nitrogen, phosphorus, potassium, and magnesium, is often used as fertilizer [3,4]. Studies have shown it lowers soil pH and improves nutrient content, with application rates ranging from 20–100 tons/ha, though optimal results are around 20–30 tons/ha [[5], [6], [7], [8], [9], [10]]. However, direct application can negatively impact soil properties, including water retention, hydraulic conductivity, and the nitrogen cycle, and may cause phytotoxicity at high concentrations. To mitigate these effects, pre-treatments such as fermentation and anaerobic digestion are used, producing biogas, biofertilizers, and bioactive polyphenols [[11], [12], [13], [14]].

Despite its potential, farmers lack awareness of proper application rates for OSW, and research on its nitrogen mineralization is limited. This dataset aims to determine the nitrogen utilization coefficient (NUC) from fresh and fermented OSW applied to calcareous soil cultivated with wheat, addressing the need for precise guidelines on OSW usage in agriculture.

## 3 Data Description

Table 1 shows the physicochemical characteristics of the soil from the college farm (Abi Jerash, Syria). The soil is clay loam soil, which is relatively aerated soil according to the bulk density and total porosity values, with slightly alkaline soil pH values. It is characterized by its high content of total carbonates, reaching 50 %. In addition, it is noted that the soil content of organic matter increased, reaching 2.80 %, and this may be due to the annual additions of organic waste to the soil. As for the soil content of fertility elements, it was characterized by an average content of total nitrogen, which amounted to 0.14 %, as well as high contents of available P and K, with values of 74.2 and 112.17 mg kg^−1^, respectively.

**Table 1:** Soil physicochemical properties.

Parameter	Units	Value
Sand	**%**	**29.8**
Silt	%	30.95
Clay	%	39.25
Soil texture (USDA classification)		Clay Loam
Bulk density	g cm^−3^	1.1
Total porosity	%	57.84
Soil pH_H2O(1:2.5)_	Adimensional	8.1
Electrical conductivity _(1: 5)_	dS m^−1^	0.45
Total carbonates	%	50
Organic matter	%	2.8
Total Nitrogen	%	0.14
Available P	mg kg^−1^	74.2
Available K	mg kg^−1^	112.17

Average values (n=3)

Table 2 shows the chemical and fertility characteristics of fresh and fermented olive solid waste. The pH decreased in fresh and fermented olive solid waste, as it was less than 7 in both. The pH levels reached in each of the olive solid waste, fresh and fermented, were 5.60 and 6.10, respectively. These values can be attributed to the acidic products of olive fruits. Besides, the fermented olive oil solid waste has moderate electrical conductivity but a low organic matter and reduced C:N ratio compared to fresh olive oil solid waste (Table 2).

**Table 2:** Some chemical and fertility properties of fresh and fermented olive oil solid waste.

Parameter		Fresh Olive Oil Solid Waste	Fermented Olive Oil Solid Waste
pH	Adimensional	5.6	6.1
EC	dS m^−1^	2.62	3.38
Organic matter	%	92.73	76.08
Organic carbon	%	53.78	44.13
N	%	1.2	1.5
P	%	0.37	0.5
K	%	0.24	4.02
C:N ratio		44.82	29.42

Average values (n=3)

This indicates that aerobically fermentation can help break or modify the organic matter in the olive oil waste, releasing macronutrients such as N, P, and K, especially available K, and therefore making them more available for plants. However, this process seems to appear to have increased the pH and electrical conductivity of the waste.

In this sense, the low C:N ratio in fermented wastes reflects the availability of plant nutrients, the formation of humic and fulvic acids, and the contribution to the modification of soil alkalinity and the liberation of mineral elements from insoluble compounds. Very high (>40:1) or reduced (<10:1) C:N ratios can give inappropriate final compost since high C:N indicates an excessive C and insufficient N for aerobic microbes, while very low ratios <10 suggest an underutilization of N and nitrogen loss, with nitrogen available and released into the environment as NH_3_ or N_2_O. In our case, an acceptable mature and stable final compost was obtained, although a C:N ratio below 20 would be preferable [[15], [16], [17]]. In this sense, more time is required to obtain appropriate compost [18].

The nutrient utilization coefficient (NUC) in the organic fertilizer is meant by the difference between the nitrogen absorbed by the plants in the organic fertilizer treatment and the nitrogen of the control plants concerning the amount of nitrogen in the added organic fertilizer. This coefficient is an indicator of the decomposition of organic fertilizers added to the soil and the degree of mineralization, and thus the rate of release of mineral nitrogen and its readiness for plants [19,20]. The percentage of the apparent utilization coefficient of nitrogen (NUC) in the fresh and fermented olive solid waste treatments showed significant differences between treatments. The NUC in fermented olive oil solid waste was 50.15 %, and this value was lower in fresh wastes, reaching 35.36 % (Table 3).

**Table 3:** Nitrogen utilization coefficient in olive oil solid waste treatments (NUC %).

Organic waste (Application rate 20 ton h^−1^)	NUC %
Fresh olive oil soil waste	35.36*b*
Fermented olive oil solid waste	50.15*a*

LSD value (5 %): 6.21

## 4 Experimental Design, Materials and Methods

### 4.1 Soil characterization

A topsoil (0–20 cm sampling depth) was collected from an agricultural area at the Abi Jerash research field (College of Agriculture, Damascus University, Syria). Several soil samples were collected from this area to get a compound sample stored in polyethylene bags. After the soil sampling and once in the laboratory, soil samples were air-dried, passed through a 2 mm sieve and homogenized before physicochemical characterization. The particle-size distribution was analyzed using the hydrometer method with the addition of a soil dispersant (sodium hexametaphosphate), while bulk and particle densities were analyzed using the cylinder and the pycnometer method, respectively. The soil pH_H2o_ and electrical conductivity (EC) were analyzed through a soil suspension in water (1:2.5 and 1:5 soil/water extract, respectively). The total Kjeldahl-N (organic plus ammonium-N) was analyzed by Kjeldahl digestion with sulphuric acid and distillation with boric acid. The available soil P was extracted by the Olsen method (NaHCO_3_ 0.5 M, pH 8.5; 1:20 soil: extractant ratio) and measured by spectrophotometer, while the available K was analyzed using a solution of ammonium acetate (1 M), with an extraction ratio of (1:10 soil: extractant ratio) and measured by Atomic Absorption Spectrometer (AAS) (Varian SpectrAA-880/GTA100 Atomic Absorption Spectrometer).

### 4.2 Olive oil solid waste

Olive oil solid waste was provided by an oil mill in the Najha area (south of Damascus, Syria) and divided into two parts. The fresh part was dried directly and preserved, and another part was aerobically fermented for three months by placing it in a bag, moistening it, and then closing the bag. After three months, both fractions were added to the soil at a rate of 20 tons ha^−1^ for the pot experiment once this amendment rate was indicated as environmentally safe [[5], [6], [7], [8], [9], [10]]. Olive solid wastes were physiochemically characterized. The pH and the EC were estimated in a 1:5 waste/water extract, the organic matter by incineration (550°C), the organic carbon by oxidation with potassium dichromate, total Nitrogen by the Kjeldahl method, the total phosphorus colourimetrically by spectrophotometer, while total potassium by incineration and measured by a flame analyzer.

### 4.3 Pot experiment

The experiment was carried out in plastic pots (the height of the pot is 15 cm, the average diameter is 20 cm, and each container can hold 2 kg). Fresh olive solid waste and fermented olive solid waste were manually mixed with soil and transferred to the pots at a rate of 20 tons h^−1^ at a rate of 24 g pot^−1^ based on the weight of one hectare of soil with a density of 1.1 g cm^−3^ and a depth of 20 cm equaling 1650 tons. A non-organically fertilized treatment (without added olive solid waste) was considered a control treatment. Pots were placed in the open air outside the laboratory (20-25 °C and 10 h light, 14 h dark). Six replicates were carried out for each treatment.

At the beginning of the assay, all treatments, including the control blank, were fertilized with phosphate and potassium solid fertilizers at the following rates: triple superphosphate 46 % P_2_O_5_ at a rate of 80 kg P_2_O_5_ h^−1^, potassium sulfate 50 % at a rate of 80 kg K_2_O h^−1^. For each pot, seven seeds equivalent to 0.24 g of durum wheat seed (*Triticum durum* cv Sham 3) were placed at a rate of 20 kg seed h^−1^. After seed germination, only five plantlets were left to grow for an additional 15 days to avoid intraspecific competition. The pots were irrigated with distilled water when the soil moisture reached 80 % of the field capacity by weighing the pots before each irrigation to reach the previous weight when planting and after the first irrigation.

### 4.4 Estimation of the coefficient of nitrogen utilization by plants

So as to estimate the availability of nitrogen in the olive solid waste and fermented olive solid waste, we followed the equation indicated by [19,20] to calculate the nitrogen utilization coefficient (Eq. (1)):

(1) $$\text{NUC}=\left(\left(N_{T}-N_{0}\right)/N_{\text{app}}\right)\times 100$$

where NUC is the Nitrogen Utilization Coefficient, N_T_ is the amount of Nitrogen in grams in plants fertilized with olive amendment, N_0_ is the amount of Nitrogen in grams in control plants, and N*app* is the amount of Nitrogen applied, calculated as follows: the amount of olive solid waste added to each pot x the percentage of Nitrogen in the dregs (dry material), which is equal to 24×1.2 / 100 = 0.288 g N pot^−1^ for the treatment of fresh olive oil solid waste, and it is equal to 24×1.5/100 = 0.36 g pot^−1^ N for fermented dregs treatment.

### 4.5 Statistical analysis

The experiment was designed according to a completely randomized design. Results from all parameters were expressed as the mean ± standard deviation (SD). After verifying the homogeneity of the variances (Levene’s test), the LSD 0.05 values for mean value comparisons were calculated using the Tukey test. Statistical procedures were carried out with IBM SPSS Statistics v23 (IBM®, USA).

## Limitations

Due to logistical and funding constraints, it is not possible for us to conduct additional experiments at this stage (more replicates, study sites and plant species examined). Nevertheless, we would like to emphasize that the current study provides novel and relevant data in a field where such information is still scarce. Our approach was carefully designed to ensure consistency and control within the limitations of available resources. While the sample size and site diversity were limited, the data collected still reveal clear patterns and trends that contribute meaningfully to understanding.

## Ethics Statement

The authors declare that they have no known competing financial interests or personal relationships that could have appeared to influence the work reported in this paper. No humans or animals have been used in this experiment. No data has been collected from social media platforms.

## CRediT authorship contribution statement

**Abd Al Karim Jaafar:** Conceptualization, Methodology, Formal analysis, Investigation, Resources, Data curation, Writing – original draft. **Akram Al-Balkhi:** Conceptualization, Methodology, Formal analysis, Investigation, Resources, Data curation, Writing – original draft. **Andrés Rodríguez-Seijo:** Data curation, Visualization, Writing – review & editing.

## Data Availability

(Mendeley Data).Effect of fresh and fermented olive oil solid waste on nitrogen utilization coefficient for crops (Original data)

## References

[1] A. El Ibrahem, M. Abdine, A. Dragotta. The olive oil sector in Syria [printed version also available. In: Di Terlizzi B., Dragotta A., Jamal M. (Eds). Syrian national strategic plan for olive oil quality: final report. Bari: CIHEAM (2007) pages 17-34 (Options Méditerranéennes: Série A. Séminaires Méditerranéens; n. 73) https://om.ciheam.org/om/pdf/a73/00800334.pdf

[2] Kavdir Y., Killi D. (2008). Influence of olive oil solid waste applications on soil pH, electrical conductivity, soil nitrogen transformations, carbon content and aggregate stability. Bioresour. Technol..

[3] Sánchez-Sánchez C., González-González A., Cuadros-Salcedo F., Cuadros-Blázquez F. (2020). Two-phase olive mill waste: a circular economy solution to an imminent problem in Southern Europe. J. Clean. Prod..

[4] Khalil J., Jaafar A.A., Habib H., Bouguerra S., Nogueira V., Rodríguez-Seijo A. (2024). The impact of olive mill wastewater on soil properties, nutrient and heavy metal availability - a study case from Syrian vertisols. J. Environ. Manag..

[5] M. Cengel, Z. Ersacan, C. Acunaz. A study on effects of fig, raisin and olive oil plant waste on microbiological mineralization of soil and organism groups Izmir, Turkey. R&D Project No: 010. 59-78 (1988).

[6] Seferoğlu S., Kılınç I I. (2002). Proceedings of the 13th Internatıonal Scientific Centre of Fertilizers (CIEC) Tokat.

[7] C. Acunaz C. A study on refinements and utilization of black water from olive press plants of Taris olive oil union Turkey. R&D project no: 012:65-78. (1987)

[8] T. Isıklı. An investigation on analytical properties of oil vegetation water derived from olive fabrics. Publications of olive research institute agricultural and rural ministry of Turkey. pp. 56-46 (1992)

[9] M. Hermoso M. Use of olive by-products as fertilizers. International course on fertilization and intensification on olive cultivation. FAO Madrid, Spain. pp. 65-88 (1983)

[10] Boz O., Dogan M.N., Albay F. (2003). Olive processing waste for weed control. Weed Res..

[11] Batuecas E., Tommasi T., Battista F., Negro V., Sonetti G., Viotti P., Fino D., Mancini G. (2019). Life cycle assessment of waste disposal from olive oil production: anaerobic digestion and conventional disposal on soil. J. Environ. Manag..

[12] Jaafar A.A.K. (2021). Effect of fresh and fermented olive solid waste and cow manure on zinc forms in calcareous soil and wheat plant productivity. Am. J. Biol. Environ. Stat..

[13] Fernández-Rodríguez M.J., Mancilla-Leytón J.M., Jiménez-Rodríguez A., Borja R., Rincón B. (2021). Reuse of the digestate obtained from the biomethanization of olive mill solid waste (OMSW) as soil amendment or fertilizer for the cultivation of forage grass (Lolium rigidum var. Wimmera). Sci. Total Environ..

[14] Tsigkou K., Sivolapenko N., Kornaro M. (2022). Thermophilic dark fermentation of olive mill wastewater in batch reactors: effect of pH and organic loading. Appl. Sci..

[15] Ibrahim G.A.Z., El-Tayeb T.S., Omar A.M., Balah M.A., Ramadan EM E.M. (2016). Effect of fermented olive mill waste water on the growth and productivity of sorghum grown under field conditions. J. Environ. Sci..

[16] Akratos C.S., Tekerlekopoulou A.G., Vasiliadou I.A., Vayenas D.V., Galanakis C.M. (2017). Olive Mill Waste.

[17] Azis F.A., Choo M., Suhaimi H., Abas P.E. (2023). The effect of initial carbon to nitrogen ratio on kitchen waste composting maturity. Sustainability.

[18] Majbar Z., Lahlou K., Abbou M., Ammar E., Triki A. (2018). Co-composting of olive mill waste and wine-processing waste: an application of compost as soil amendment. J. Chem..

[19] Bousselhaj K., Fars S., Laghmari A., Nejmeddine A., Ouazzani N., Ciavatta C. (2004). Nitrogen fertilizer value of sewage sludge co-composts. Agronomie.

[20] Kuziemska B., Trębicka J., Wysokinski A. (2021). Uptake and utilization of nitrogen from organic fertilizers influenced by different doses of copper. Agronomy.

